# Alectinib-Loaded Chitosan–Alginate Nanoparticles: A Novel Synthesis Method with In Vitro and In Vivo Evaluations

**DOI:** 10.3390/pharmaceutics17040492

**Published:** 2025-04-08

**Authors:** Tha’er Ata, Israa Al-Ani, Nida Karameh, Mahmood R. Atta, Wael Abu Dayyih

**Affiliations:** 1 Department of Pharmaceutics and Pharmaceutical Technology, Faculty of Pharmacy, Pharmacological and Diagnostic Research Center (PDRC), Al-Ahliyya Amman University, Amman 19328, Jordan; mahmoodraad103@gmail.com; 2 Department of Pharmacology and Clinical Pharmacy, Faculty of Pharmacy, Middle East University, Amman 11831, Jordan; n.karameh@meu.edu.jo; 3 Department of Pharmaceutical Chemistry, Faculty of Pharmacy, Mutah University, Al Karak 61710, Jordan; wabudayyih@mutah.edu.jo

**Keywords:** Alectinib, chitosan, alginate, nanoparticles, ion gelation, bioavailability

## Abstract

**Background/Objectives**: Non-small cell lung cancer (NSCLC) constitutes over 84% of all lung cancer cases and is a leading cause of cancer-related mortality globally. Alectinib, a second-generation anaplastic lymphoma kinase (ALK) inhibitor, is effective in ALK-positive NSCLC; however, its clinical potential is hampered by poor aqueous solubility and limited oral bioavailability. This study aimed to develop Alectinib-loaded chitosan–alginate nanoparticles (ACANPs) to enhance its solubility, oral bioavailability, and therapeutic efficacy. **Methods**: ACANPs were synthesized using a novel combined solid/oil/water (s/o/w) emulsification technique with ionotropic gelation. Characterization was performed using Fourier-transform infrared spectroscopy (FTIR), differential scanning calorimetry (DSC), dynamic light scattering (DLS), and zeta potential measurements. A validated high-performance liquid chromatography (HPLC) method quantified the Alectinib. In vitro drug release studies compared free Alectinib with ACANPs. Cytotoxicity against NSCLC cell lines (A549 and H460) was assessed using MTT assays. Pharmacokinetic parameters were evaluated in rats using LC–MS/MS. **Results**: ACANPs showed a high encapsulation efficiency (~97%), an average particle size of 161 nm, and a positive zeta potential of +21 mV. In vitro release studies revealed a threefold increase in drug release from ACANPs over 48 h compared to free Alectinib. Cytotoxicity assays demonstrated significantly reduced IC_50_ values for ACANPs. Pharmacokinetic analyses showed an enhanced maximum plasma concentration (C_max_) and area under the curve (AUC), indicating a 78% increase in oral bioavailability. **Conclusions**: ACANPs substantially improved the solubility, cytotoxic efficacy, and oral bioavailability of Alectinib, suggesting their potential as a promising nanocarrier system for enhancing NSCLC treatment outcomes.

## 1. Introduction

Non-small cell lung cancer (NSCLC) accounts for more than 84% of all lung cancer cases and continues to be a primary cause of cancer-related mortality globally [1,2]. Commonly diagnosed in adults aged over 65, it includes various histological subtypes: adenocarcinoma, squamous cell carcinoma, and large cell carcinoma [3]. Smoking is the primary risk factor, alongside exposure to environmental toxins [4].

**Figure 1:**
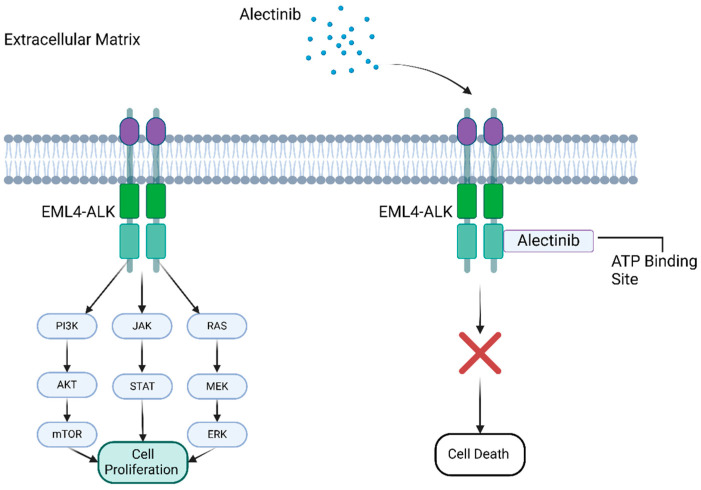
The inhibitory role of Alectinib on EML4-ALK-positive NSCLC.

A fundamental aspect of current NSCLC treatments is the targeting of oncogenic drivers, such as EGFR and KRAS mutations, and ALK gene rearrangements [1]. Alectinib, a second-generation ALK tyrosine kinase inhibitor (TKI), has demonstrated notable efficacy in patients with ALK-rearranged NSCLC, including those resistant to the first-generation ALK inhibitor crizotinib [5]. Alectinib demonstrates enhanced central nervous system (CNS) penetration, effectively addressing a common resistance mechanism, as approximately 50% of ALK-positive patients develop brain metastases [6,7,8]. The clinical advantages of Alectinib led to its approval by the U.S. Food and Drug Administration (FDA) for metastatic ALK-positive NSCLC in 2015, with an expanded indication for adjuvant therapy in 2024 [9,10]. However, insufficient aqueous solubility and poor oral bioavailability persist as major issues. Figure 1 shows the mechanism of action of Alectinib.

Nanoparticle-based systems present an attractive way to overcome these limitations. Chitosan, a biopolymer sourced from chitin, possesses several advantages: it is biocompatible, biodegradable, and demonstrates mucoadhesive characteristics that can improve drug stability and absorption [11,12]. Positively charged amino groups facilitate ionic crosslinking with anionic polymers such as alginate, which is sourced from brown seaweed and rapidly forms gels in the presence of divalent cations [13,14]. The pH-sensitive release profile of alginate and the mucoadhesive properties of chitosan can collaborate to enhance encapsulation efficiency and control drug release, as well as improve oral bioavailability.

This study demonstrates the development of Alectinib-loaded chitosan–alginate nanoparticles (ACANPs) by a novel approach that combines ionotropic gelation with solid/oil/water emulsification. Researchers hypothesized that encapsulating Alectinib in chitosan–alginate nanostructures will enhance its aqueous solubility, improve its therapeutic activity against NSCLC cells, and enhance oral bioavailability. The goals of this study encompassed (i) improving the nanoparticle production method to achieve an optimal encapsulation efficiency and particle size, (ii) determining drug release profiles under physiologically relevant conditions, (iii) evaluating cytotoxicity against NSCLC cell lines, and (iv) assessing pharmacokinetic parameters in an animal model. This study seeks to enhance Alectinib delivery techniques for the better management of NSCLC.

## 2. Materials and Methods

### 2.1. Materials

This study utilized a variety of reagents and materials essential for its experimental procedures. Alectinib hydrochloride was obtained from (Biosynth Zurich, Switzerland, distributed by Cymit Química S.L., Barcelona, Spain) while dimethyl sulfoxide (DMSO) was sourced from EMSURE^®^ in Grove Village, IL, USA. Methanol (HPLC grade) and acetonitrile (ACN) were supplied by Honeywell International, Charlotte, NC, USA. Ultra-purified de-ionized water was procured from AAU/PDRC in Jordan. Additional components such as polyethylene glycol (PEG), Tween 80, Cremophor RH 40, low-molecular-weight chitosan, Pluronic F127, and lecithin were acquired from Sigma-Aldrich, St. Louis, MO, USA. This study also employed Roswell Park Memorial Institute (RPMI-1640) medium and MTT reagent from Thermo Fisher Scientific, Waltham, MA, USA, trypsin–EDTA from Euro Clone in Pero, Italy, and crystal violet from Merck Millipore, Burlington, MA, USA. The biological materials included A549 and H460 lung cancer cell lines, which were obtained from the American Type Culture Collection in Manassas, VA, USA, and phosphate-buffered saline (PBS) sourced from Sigma-Aldrich, USA. Formic acid was procured from Honeywell International, and sunflower oil (99.5%) was sourced locally in Jordan. These high-grade reagents and materials played a critical role in ensuring the precision and reliability of the study’s outcomes.

### 2.2. UV–Vis Spectroscopy of Alectinib, CANPs, and ACANPs

A UV–Vis spectrophotometer (Model U-2000, Hitachi, Tokyo, Japan) was utilized to determine the maximum absorbance wavelength (λ_max_) of free Alectinib and blank chitosan–alginate nanoparticles (CANPs). For sample preparation, two milligrams of Alectinib was dissolved in 10 mL of methanol to create the stock solution. The measurement involved scanning the prepared samples from 200 to 800 nm, using a quartz cuvette to identify the λ_max_ and assess any potential interference. This meticulous procedure ensured the accurate UV detection of Alectinib, while confirming that the absorption peaks of the nanoparticle excipients did not overlap significantly with the Alectinib absorption peak.

### 2.3. Development and Validation of HPLC Method for Alectinib Determination in Formulation

An HPLC–UV method was optimized and validated for quantifying Alectinib in formulations. A Macherey-Nagel 100-5 C18 ec 150 × 4.6 mm column, coupled with Shimadzu LabSolutions software DB version 6.5, was used for data processing.

#### 2.3.1. Chromatographic Conditions

The mobile phase for the analysis consisted of methanol, acetonitrile, and water in a 40:30:30 (*v*/*v*) ratio, delivered at a flow rate of 1.0 mL/min. The detection wavelength was set at 340 nm, determined from UV scans, with an injection volume of 10 μL. The aqueous phase was prepared using 500 mL of HPLC-grade water, with its pH adjusted to 3.6 by adding one drop of 85% orthophosphoric acid. This was combined with 150 mL of acetonitrile and 200 mL of methanol. All components were filtered through a 0.22 μm nylon membrane and sonicated for 10 min to de-gas before use, ensuring optimal chromatographic performance.

#### 2.3.2. Method Validation

Linearity, precision, accuracy, selectivity, specificity, and recovery were evaluated according to ICH guidelines to validate the analytical methods used in the study. Linearity was assessed using 10 concentration points ranging from 0.195 to 100.0 μg/mL, prepared by serially diluting a 0.2 mg/mL stock solution of Alectinib in methanol. The peak area under the curve (AUC) was plotted against concentration, with linearity accepted at R^2^ > 0.99. Precision and accuracy were determined using three quality control (QC) concentrations—QC High (90 μg/mL), QC Medium (50 μg/mL), and QC Low (5 μg/mL)—each analyzed in six replicates. Precision, expressed as RSD, was required to be <2%, while accuracy had to fall within 90–110% of the theoretical values. Selectivity and specificity were confirmed by comparing chromatograms of blank CANPs, mobile-phase, and Alectinib samples, ensuring no interference near the retention time of Alectinib. Recovery was evaluated at three concentrations (5, 50, and 90 μg/mL) using Alectinib-loaded nanoparticles. The nanoparticles were vortexed in a 1:1 methanol–acetonitrile solution, sonicated, and centrifuged to release the encapsulated drug, which was quantified by HPLC–UV. Recovery was validated with a target RSD < 2% and values between 85–115%, according to ICH guidelines.

### 2.4. Preparation of Alectinib-Loaded Chitosan–Alginate Nanoparticles (ACANPs)

Alectinib-loaded nanoparticles were synthesized employing ion gelation, using sodium tripolyphosphate (TPP) as the crosslinking agent [15]. Initial investigations used a 1:1 chitosan-to-TPP ratio; however, excessive reactivity and a high gelation led to gradual adjustment from 1:1 to 5:1 to improve the gelation kinetics and crosslinking efficiency [16]. To increase nanoparticle dispersion and ionic strength, NaCl was tested [17]. However, it affected the particle stability, so it was removed from the formulation. Alectinib was solubilized in 5 mg/mL DMSO for better solubility. DMSO–methanol (1:1), ethanol, and distilled water were tested to increase solubility and decrease drug precipitation during crosslinking. To improve the nanoparticle stability and lipophilicity, sodium alginate was used as a secondary crosslinker [18]. Comprehensive chitosan–alginate ratio testing examined nanoparticle uniformity and stability. Chitosan–alginate ratios of 1:1, 1:2, 1:3, 1:4, and 1:5 were tested to determine the effects of high alginate concentrations. In contrast, ratio methods examined chitosan-dominant formulations at 2:1, 3:1, 4:1, and 5:1 chitosan–alginate ratios. For a high nanoparticle stability and crosslinking efficiency, a 1:1:5 TPP:ALG:CS dual crosslinking agent system was adopted.

**Figure 2:**
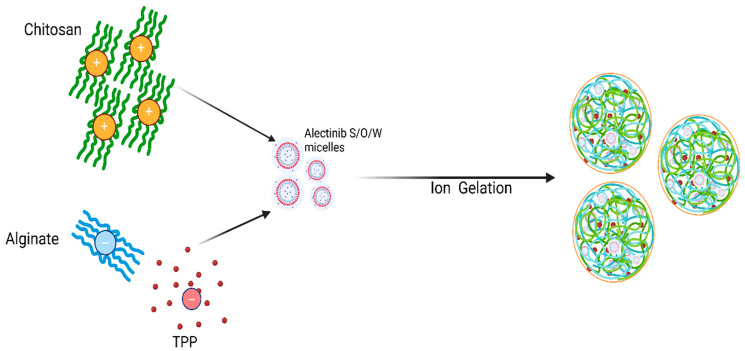
A simplified illustration of the novel method for ACANP preparation.

Our novel nanoparticle synthesis approach combines the ion gelation method and solid/oil/water (s/o/w) emulsification technique [19]. This method eliminates the organic solvent’s negative effect on the crosslinking process and toxicity, while improving the nanoparticle quality and encapsulation efficiency, as illustrated in Figure 2.

**Figure 3:**
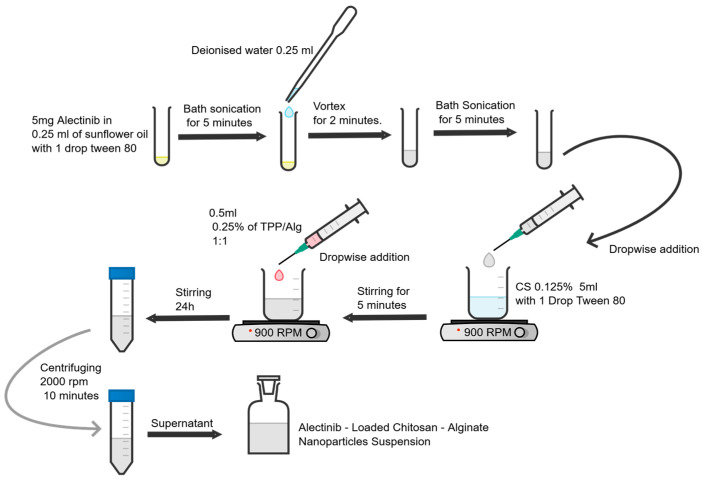
The synthesis process of Alectinib-loaded chitosan–alginate nanoparticles.

A 0.125% (*w*/*v*) chitosan solution in 1% acetic acid is prepared first. Sodium acetate is required for adjusting the pH to 4.0–5.0 for nanoparticle fabrication. One drop of Tween 80 in the chitosan solution improves dispersion and prevents agglomeration. Alectinib (5 mg) is then dispersed in 0.25 mL sunflower oil with one drop of Tween 80 and sonicated for 5 min to ensure uniformity. Next, 0.25 mL of deionized water is added, and the mixture is vortexed and sonicated to generate a stable emulsion. This emulsion is gradually introduced into the chitosan solution during stirring at 900 rpm. Tripolyphosphate (TPP) and sodium alginate are added in a 1:1:5 ratio to initiate ionic gelation and nanoparticle production. The final mixture is stirred at 900 rpm for 24 h to guarantee homogeneity and particle size uniformity. After stirring, the suspension is centrifuged at 2000× *g* rpm for 10 min to eliminate any unreacted materials. Amber glass containers are used to store the finished product at room temperature, preventing light-induced degradation. Figure 3 summarizes the synthesis process.

### 2.5. Optimization of Formulation

Our novel approach to prepare Alectinib-loaded chitosan–alginate nanoparticles focused on maximizing drug-loading efficiency and ensuring formulation stability by optimizing the selection and ratios of crosslinking agents and surfactants.

### 2.6. Characterization of Alectinib-Loaded Nanoparticles

#### 2.6.1. Particle Size (PS), Polydispersity Index (PDI), and Zeta Potential

Dynamic light scattering (DLS) was used to measure average particle size, PDI, and zeta potential with a Malvern Zetasizer (UK). Analyses were performed at 25 °C in triplicate, and the results were reported as mean ± standard deviation (SD) [20,21]

#### 2.6.2. Fourier-Transform Infrared (FTIR) Spectroscopy

FTIR spectra were obtained (400–4000 cm^−1^) using a Shimadzu IR-Prestige 21 spectrometer to detect potential chemical interactions among Alectinib, chitosan, alginate, and the whole complex. Samples were mixed with KBr and pressed into discs [22].

#### 2.6.3. Differential Scanning Calorimetry (DSC)

Thermal properties and potential interactions were evaluated with DSC (DT-60, Shimadzu, Tokyo, Japan). Alectinib powder, chitosan, alginate, and ACANPs (F13) were heated from 4 to 300 °C at 10 °C/min to detect shifts in melting peaks or glass-transition events [23].

#### 2.6.4. Encapsulation Efficiency (EE%)

Encapsulation efficiency was determined by dissolving the dried nanoparticles in a 1:1 methanol–acetonitrile mixture and quantifying Alectinib at 340 nm via the developed HPLC method. EE% was computed according to Equation (1) [24].

(1) $$EE\%=\frac{Amount\;of\;drug\;in\;NPs*100}{Amount\;of\;total\;drug\;}$$

#### 2.6.5. Stability Assessment

The ACANPs were monitored for three months by measuring particle size, PDI, and zeta potential to evaluate potential changes in colloidal stability. Suspension samples were stored at room temperature, protected from light, and tested at set intervals with the Malvern Zetasizer software (version 8.03).

### 2.7. In Vitro Drug Release 

**Figure 4:**
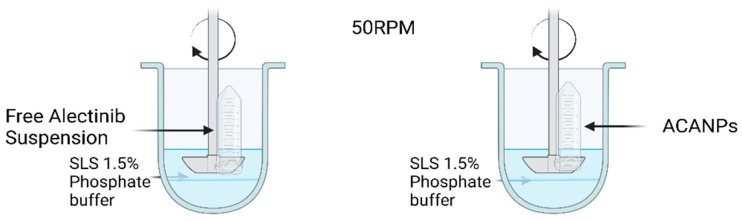
Schematic illustration of the release method.

An in vitro drug release study was performed to quantify the drug released from the ACANPs vs. the Alectinib powder dispersed in water. Phosphate buffer solutions (PBS) with a pH of 6.8 were prepared for this experiment. According to a prior study, 1.5% SLS was incorporated into the release media to maintain sink conditions [25]. The conditions for dissolution were as follows: dissolution apparatus USP II (paddle), stirring speed of 50 rpm, temperature of 37.0 ± 0.5 °C, and release media volume of 200 mL. Samples were positioned in a plastic tube encased with a dialysis membrane featuring a molecular weight cut-off (MWCO) of 14,000 Da, which had been previously immersed in deionized water for 24 h [26]. A sample of the nanoparticles, corresponding to 5 mg of Alectinib, was employed for the release investigation. Samples of 0.5 mL were collected at designated time intervals (15 min, 1, 2, 3, 4, 5, 6, 8, 24, and 48 h), filtered through a 0.22 µm membrane, and subsequently fed into the HPLC system at a wavelength of 340 nm. Figure 4 illustrates the release method.

### 2.8. In Vitro Cytotoxicity (MTT Assay)

This study employed A549 and H460 lung cancer cell lines, widely used in cancer research, to evaluate the efficacy of ACANPs and Alectinib. Cells were cultured in RPMI medium supplemented with L-glutamine, fetal bovine serum, and penicillin-streptomycin under standardized conditions. After thawing them from liquid nitrogen storage, the cells were processed in a laminar flow hood, centrifuged to remove cryoprotectants, and resuspended in fresh medium before being seeded in T-25 flasks. Subculturing was performed when the cells reached 80–90% confluency, using trypsin–EDTA for detachment, and viability was assessed via Trypan blue staining. For treatment, 5000 cells per well were seeded in 96-well plates and treated with varying concentrations of ACANPs and Alectinib (80–3.125 µg/mL) for 72 h, with controls included for comparison. The MTT assay was conducted to evaluate cell viability, where formazan crystals were solubilized in DMSO, and absorbance was measured at 590 nm. The IC_50_ values were calculated using Equation (2) to assess growth-inhibitory effects, revealing the compounds’ efficacy against A549 and H460 cell lines [27].

(2) $$\mathrm{Cell}\;\mathrm{survival}\;\mathrm{rate}=\frac{\;(1-\left(\mathrm{ABS}-\mathrm{MIN}\right))}{\left(\mathrm{MAX}-\mathrm{MIN}\right)}\times 100\%$$

### 2.9. Pharmacokinetic Investigation and Bioavailability Assessment

#### 2.9.1. LC–MS/MS

Samples were analyzed using an Agilent 1200 HPLC coupled with a SCIEX 4000 mass spectrometer (AB Sciex Pte. Ltd., Singapore). Chromatographic separation was carried out on an ACE C8 column with a mobile phase of 65% water, 35% acetonitrile, and formic acid (1.0 mL/L), at a 1.0 mL/min flow rate. The parameters are presented in Table S1.

#### 2.9.2. Method Development

The analytical method for determining Alectinib in rat plasma was validated in accordance with FDA guidelines for bioanalytical method validation, ensuring its reliability for pharmacokinetic studies. The validation process involved assessing key parameters, including linearity, precision, accuracy, sensitivity, selectivity, and recovery. Linearity was evaluated using a calibration curve created from eight standard concentrations of Alectinib (10–4000 ng/mL), with a target correlation coefficient (R^2^) of ≥0.98. Precision and accuracy were assessed at four concentration levels: LLOQ (10 ng/mL), QC Low (30 ng/mL), QC Medium (1500 ng/mL), and QC High (3500 ng/mL). Intra-day and inter-day precision, expressed as RSD, met the acceptance thresholds of <15% for all levels, except LLOQ, which allowed up to 20%. Accuracy was confirmed within the range of 85–115%, with LLOQ variations permitted up to 20%. Selectivity and specificity were demonstrated by analyzing blank plasma samples to ensure no interference at the retention times of Alectinib and the internal standard, Rosuvastatin, with matrix signals below 5% of the internal standard’s peak area. Recovery was evaluated at three quality control levels (30, 1500, and 3500 ng/mL), comparing the mean peak areas of extracted plasma samples to clean standard solutions.

#### 2.9.3. Animal Handling and Ethics

All animal protocols were approved by the Institutional Ethical Committee, Faculty of Pharmacy, Al-Ahliyya Amman University, Amman, Jordan (IRB AAU1/13/2023–2024). Male Wistar rats (weighing 0.250 ± 0.020 kg, *n* = 12) were housed under standard conditions with unrestricted access to water and subjected to overnight fasting prior to dosing. The rats were divided into two groups: Group 1 (*n* = 6) received Alectinib-loaded chitosan–alginate nanoparticles (ACANPs) containing the equivalent of 5 mg Alectinib per rat via oral gavage, while Group 2 (*n* = 6) was administered free Alectinib (5 mg per rat) in a water suspension.

#### 2.9.4. Dosing and Blood Sampling

Tail vein blood samples (~350 μL) were collected at 0, 0.5, 1.5, 2, 3, 4, 6, 8, 10, 24, 48, and 72 h. Samples were centrifuged, and the plasma was stored at −80 °C until LC–MS/MS analysis.

#### 2.9.5. Non-Compartmental Pharmacokinetic Analysis

Alectinib plasma concentrations (ng/mL) were plotted over time, and non-compartmental analysis (NCA) was conducted using Phoenix WinNonlin 8.3. Key parameters included maximum plasma concentration (C_max_), time to peak concentration (T_max_), area under the curve (AUC), elimination rate constant (Kel), and clearance (CL). Bioavailability enhancements were statistically assessed by ANOVA (90% CI), according to FDA guidance.

#### 2.9.6. Sample Preparation

Sample preparation was performed using a simple liquid–liquid extraction (LLE) method. Alectinib plasma samples were defrosted at room temperature and vortexed well. Amounts of 300 μL of Alectinib plasma sample, 500 μL of diluent (mobile phase–acetonitrile–ACN), and 50 μL of Rosuvastatin (internal standard) were added to a microcentrifuge tube. The mixture was vortexed for 2 min to ensure uniform mixing, then centrifuged at 4000 rpm for 15 min. Following centrifugation, 200 μL of the supernatant was carefully transferred into HPLC insert vials. Finally, 10 μL of the prepared sample was injected into the LC–MS/MS system for analysis.

### 2.10. Statistical Analysis

Data were evaluated using a one-way or two-way Analysis of Variance (ANOVA) to compare particle size, encapsulation efficiency, release profiles, and pharmacokinetic parameters among the tested formulations. Post hoc analyses were performed as appropriate. Significance was set at *p* < 0.05, and all methods complied with ICH and FDA guidelines for method validation and statistical reporting.

## 3. Results and Discussion

### 3.1. Development and Validation of Alectinib Analytical Methods

#### 3.1.1. UV–Vis Scan for Alectinib

An initial UV–Vis screening determined the maximum absorbance (λ_max_) of Alectinib at 340 nm in methanol (25 µg/mL). Blank chitosan–alginate nanoparticles (CANPs) showed no absorbance at 340 nm, indicating no matrix interference (Figure S1A,B).

#### 3.1.2. HPLC Method

**Figure 5:**
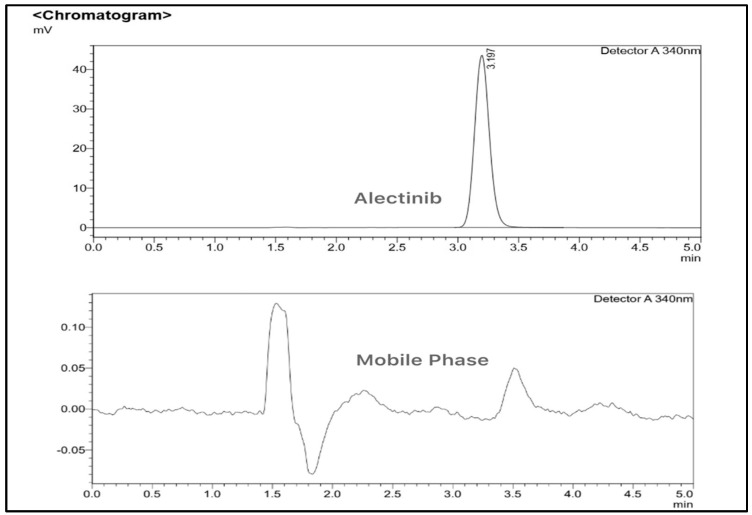
The chromatogram of Alectinib 12.5 µg/mL solution in methanol and the chromatogram of the mobile phase.

The high-performance liquid chromatography (HPLC) method with UV detection at 340 nm was optimized using Shimadzu LabSolutions software DB version 6.5. The chromatographic analysis of 12.5 µg/mL Alectinib in methanol and the chromatogram of the mobile phase are shown in Figure 5.

##### Lower Limit of Quantification and Lower Limit of Detection

The LOQs were determined to be 0.05082 µg/mL and 0.154 µg/mL, respectively; however all the tested concentrations remained within the validated linear range.

##### Linearity

**Figure 6:**
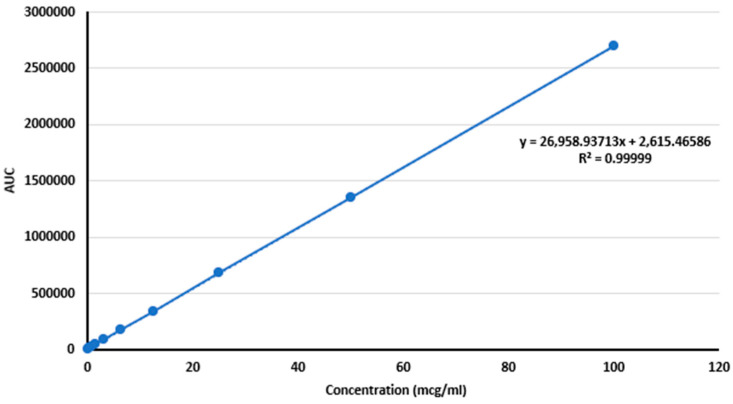
Linearity and calibration curve of ALB, showing a correlation coefficient of 0.99999.

The method validation adhered to ICH Q2(R2) guidelines and included linearity, precision, accuracy, detection and quantification limits, selectivity, specificity, and recovery. Linearity was established across 0.195–100,000 µg/mL with an R^2^ of 0.99999, and calibration data showed relative standard deviations (RSDs) under 2% (Figure 6, Table S2). Figure 6 show the linearity and calibration curve of ALB, showing a correlation coefficient of 0.99999.

##### Precision and Accuracy

The precision of the method was evaluated by analyzing three quality control (QC) samples at different concentrations—QC High (90 µg/mL), QC Medium (50 µg/mL), and QC Low (5 µg/mL) in six replicates. Precision was expressed as the relative standard deviation (RSD). The accuracy was evaluated by comparing the actual amounts recovered from control samples to their theoretical values. ICH guidelines state that the acceptance criteria require a relative standard deviation (RSD) of less than 2% for precision and an accuracy within the range of 90–110% (Table S3).

##### Selectivity and Specificity 

Selectivity and specificity were confirmed by comparing chromatograms of three samples: the blank chitosan–alginate nanoparticles, the mobile phase, and the extracted Alectinib. No interfering peaks were observed at the retention time of Alectinib in either the blank nanoparticle or mobile-phase samples, confirming the method’s ability to selectively detect and quantify Alectinib without interference from formulation components or solvents.

##### Recovery

Recovery rates for Alectinib from ACANPs ranged between 96.7 and 101.2% using a 1:1 methanol–acetonitrile solvent, with RSD values under 2%, demonstrating consistent and reliable recovery across all concentrations (Table S4).

### 3.2. Formulation and Optimization of Nanoparticles

The encapsulation efficiency (EE%) of Alectinib in chitosan nanoparticles was evaluated as a critical parameter in optimizing the formulation. Alectinib, a BCS Class IV lipophilic drug, exhibits poor solubility in aqueous systems and limited miscibility in polar solvents such as dimethyl sulfoxide (DMSO), ethanol, or methanol [25]. To improve encapsulation without compromising nanoparticle stability, a modified ion gelation technique was employed. Formulation parameters such as the type of solubilizing agent, the chitosan-to-crosslinker ratio, and the method of preparation were systematically varied (Table 1).

In early formulations (F1–F3), the use of DMSO and varying chitosan-to-TPP ratios yielded a low EE%, highlighting the limitations of solvent-only approaches. The incorporation of sodium lauryl sulfate (SLS), an anionic surfactant, in F4 also failed to improve the EE%, likely due to electrostatic repulsion between the negatively charged sulfate group and the negatively charged alginate–TPP system, leading to poor drug entrapment.

Conversely, non-ionic surfactants such as polyethylene glycol (PEG 400), Cremophor RH 40, and Tween 80 demonstrated improved encapsulation. Cremophor RH 40, a polyoxyethylated castor oil derivative, has a bulky hydrophilic polyoxyethylene chain and a lipophilic core, facilitating the solubilization of hydrophobic drugs via micelle formation. Increasing its concentration from three to five drops (F6–F8) correlated with incremental increases in EE%, indicating a dose-dependent enhancement in solubilization and drug–polymer affinity.

Similarly, Tween 80 (polyoxyethylene sorbitan monooleate), another non-ionic surfactant with a hydrophilic–lipophilic balance (HLB) value of ~15, significantly improved the EE% in F10 and F11. Its ability to form stable emulsions and promote the dispersion of lipophilic drugs into the aqueous polymer matrix likely contributed to these results. Notably, F10 and F11 achieved an EE% of 42 ± 5% and 43 ± 3%, respectively, surpassing all prior formulations.

Further enhancement was achieved through the adoption of the solid/oil/water (S/O/W) emulsification technique combined with ion gelation in F12 and F13. This hybrid approach improved the dispersion of the drug within the matrix before crosslinking, facilitating greater entrapment. F13, which also incorporated alginate as a secondary crosslinker, achieved the highest EE% of 97.1 ± 2% (*p* < 0.05). Alginate likely contributed to an improved matrix rigidity and drug entrapment through ionic interactions.

These results collectively highlight that the molecular structure and HLB of the surfactants play a critical role in the solubilization of Alectinib and its subsequent encapsulation efficiency. Non-ionic surfactants with amphiphilic architecture (e.g., Tween 80, Cremophor RH 40) are more effective than ionic surfactants like SLS due to their compatibility with both the hydrophobic drug and the polymeric matrix.

### 3.3. Characterization of ACANPs (F13)

#### 3.3.1. Results of FTIR Spectroscopy

**Figure 7:**
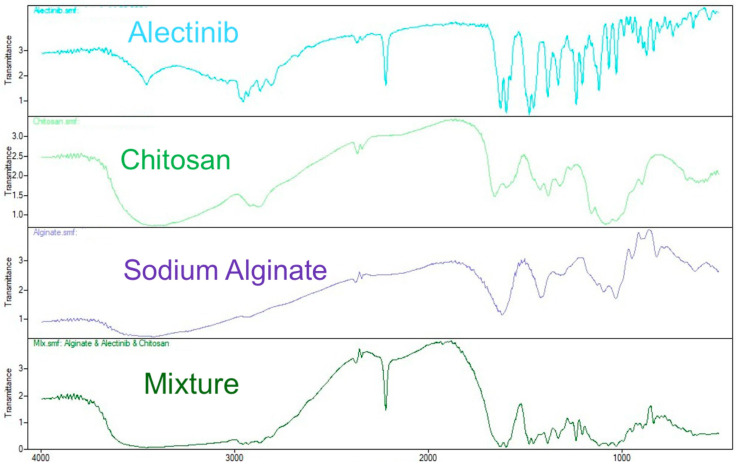
The FTIR spectra of Alectinib, chitosan, alginate, and their mixture.

An FTIR analysis was conducted to investigate potential interactions between Alectinib and the polymers chitosan and alginate used in nanoparticle formulation. The overlaid FTIR spectra of all the components and their physical mixture are presented in Figure 7.

Alectinib exhibited distinct peaks at 3454.51 cm^−1^ (N–H stretching), 3037.80 cm^−1^ (C–H aromatic stretching), 2220 cm^−1^ (C≡N stretching), and ~1608 cm^−1^ (C=C stretching). Chitosan displayed a broad band near 3400 cm^−1^ (O–H/N–H), a strong amide I peak at 1651 cm^−1^, and one for amide II near 1560 cm^−1^, along with C–O–C stretching vibrations in the 1150–1060 cm^−1^ range. Alginate showed typical carboxylate group bands at 1620 cm^−1^ (asymmetric) and 1417 cm^−1^ (symmetric), as well as C–O vibrations near 1028 cm^−1^.

In the physical mixture, most of the characteristic peaks were retained, with only minor shifts. The C≡N band of Alectinib shifted slightly to 2118 cm^−1^, and some overlapping was observed between the amide I band of chitosan and the asymmetric COO^−^ stretch of alginate (~1627 and 1597 cm^−1^, respectively). However, no significant new peaks or substantial shifts were detected, suggesting the absence of strong chemical bonding.

For clarity, the peak positions and changes are summarized in Table 2.

#### 3.3.2. Results of Differential Scanning Calorimetry (DSC)

**Figure 8:**
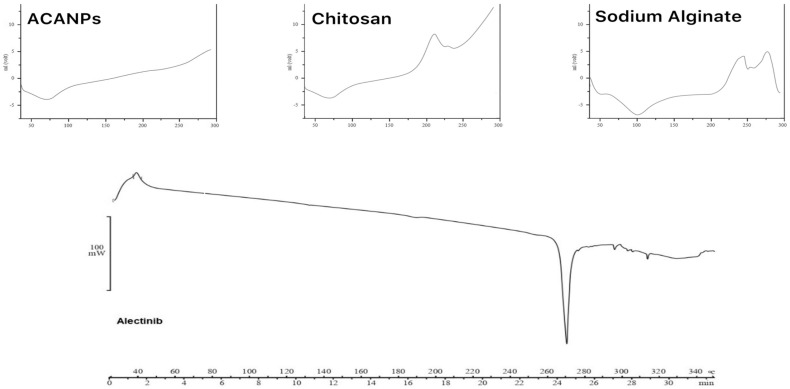
DSC thermograms of pure Alectinib, chitosan, alginate, and ACANPs showing changes in thermal behavior, indicating drug encapsulation and transformation into the amorphous state.

Differential scanning calorimetry (DSC) was performed to analyze the thermal behavior of the ACANP complex and to confirm the successful formulation of the nanoparticle system. The thermograms of individual components—Alectinib, chitosan, and alginate—along with the ACANP complex are shown in Figure 8.

Pure Alectinib displayed a sharp endothermic peak at approximately 270 °C, confirming its crystalline nature. Chitosan exhibited a broad endothermic peak between 60 °C and 75 °C, primarily attributed to the loss of bound moisture. Additionally, an exothermic transition observed above 200 °C may reflect the onset of thermal degradation rather than a true glass transition, which is often difficult to define clearly in biopolymers like chitosan [28]. Alginate demonstrated a broad endothermic region between 50 °C and 150 °C, associated with moisture loss and possible amorphous relaxation processes, consistent with its known amorphous characteristics [29].

The DSC thermogram of the ACANP complex revealed the disappearance of Alectinib’s characteristic melting peak, indicating a loss of crystallinity and suggesting successful conversion into an amorphous form within the chitosan–alginate matrix. This transformation implies the molecular dispersion of Alectinib in the polymeric network. Furthermore, the absence of new thermal events in the ACANP thermogram suggests no chemical interaction among the components, confirming their physical compatibility [30].

#### 3.3.3. Results of Particle Size, Polydispersity Index, and Zeta Potential Measurements

Dynamic light scattering (DLS) measurements, as summarized in (Table 3, Figure S10), revealed that the Alectinib-loaded chitosan–alginate nanoparticles (ACANPs; formulation F13) exhibited a mean particle size of 161.1 ± 1.5 nm, a polydispersity index (PDI) of 0.233 ± 0.013, and a zeta potential of +21.0 ± 0.32 mV (Figure S2). In comparison, the unloaded or blank chitosan–alginate nanoparticles (CANPs) displayed a smaller average particle size of 128.4 ± 3.2 nm, a comparable PDI of 0.229 ± 0.011, and a lower zeta potential of +10.0 ± 0.3 mV. These findings suggest that drug incorporation slightly increased the nanoparticle size, likely due to the entrapment of Alectinib within the polymeric matrix.

Following lyophilization, the ACANPs maintained a similar particle size (161.9 ± 2.7 nm); however, the PDI increased to 0.296 ± 0.01, indicating a moderate elevation in size heterogeneity. Notably, the zeta potential decreased to +7.86 ± 0.4 mV post lyophilization, which may be attributed to surface charge neutralization effects induced by the freeze-drying process.

#### 3.3.4. Stability of the ACANPs

Over the 90-day evaluation period, the ACANPs exhibited minimal variations in critical physicochemical parameters, indicating excellent physical stability. The particle size remained within a narrow range of approximately 156.2 to 166.6 nm, while the zeta potential values showed minor fluctuations between +20.9 and +22.9 mV. Similarly, the polydispersity index (PDI) consistently remained below 0.3, with recorded values ranging from 0.219 to 0.248. These findings (Table 4) demonstrate the absence of significant particle aggregation or instability phenomena.

The stability study was conducted under ambient storage conditions at 25 °C in dark glass containers, minimizing the potential effects of thermal or photodegradation. The maintenance of a consistent particle size and zeta potential under these conditions indicates effective electrostatic repulsion among the nanoparticles, which is essential to prevent agglomeration and sedimentation. Furthermore, the low and stable PDI values reflect the homogeneity of the formulation, a key attribute for ensuring reproducibility and safety in nanoscale drug delivery systems.

### 3.4. Results of In Vitro Drug Release 

**Figure 9:**
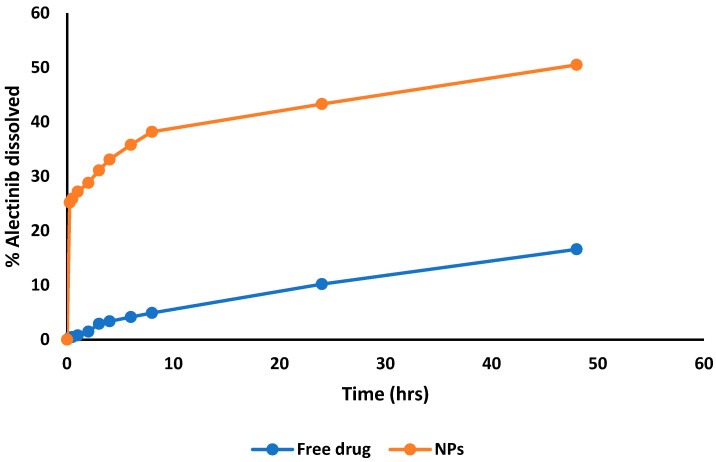
An in vitro release plot of Alectinib at 6.8 pH from the ACANPs and the free drug.

The Phase 1 release is described by a fast release pattern, which can be attributed to the biodegradability of alginate or the weak interaction between Alectinib molecules and the surface of the nanoparticles [31,32]. In the first 30 min, the ACANPs released approximately 25.2% ± 0.1%. In Phase 2, the release slows down. The cumulative release from the ACANPs over 24 h was 43.3% ± 0.1%, compared to 10.2% ± 0.2% for the free drug, demonstrating approximately a 4.2-fold increase in dissolution. At 48 h, the ACANPs released 50.5% ± 0.04%, vs. 16.6% ± 0.1% for the free drug, showing an about 3.0-fold increase. Overall, the release pattern indicates that ACANPs could provide a steady state of the drug in the body (Figure 9).

The analysis of drug release data (Table 5) reveals that the first-order and Higuchi models most effectively describe the release kinetics of ACANPs, as indicated by their high R^2^ values. The first-order model reflects a release pattern characterized by an initial rapid release followed by a slower, sustained release phase. This behavior is attributed to the rapid diffusion of the drug from the polymeric layer into the bulk medium, followed by a gradual release through an extended diffusion pathway within the polymer matrix. The Higuchi model, showing a strong correlation, mechanistically explains the drug release from the matrix system, which consists of oil droplets encapsulated within a chitosan–alginate network. This model suggests a biphasic release mechanism: an initial dissolution and partitioning phase at the interface, facilitated by surfactants reducing interfacial tension, and a subsequent diffusion-dominated phase where the drug traverses the polymeric matrix. This release mechanism aligns closely with the theoretical framework of the Higuchi model. For Alectinib powder suspended in water, the zero-order model provided the best fit, indicating a uniform drug dissolution rate over time. Additionally, the free drug data exhibited a significant correlation with the Hixson–Crowell model, which describes the gradual dissolution of powdered drugs as the particle size decreases. Due to Alectinib’s hydrophobicity, only 16.6% of the drug dissolved after 48 h, leading to the observed zero-order release profile.

### 3.5. Assessment of Cell Culture Viability (MTT Assay)

**Figure 10:**
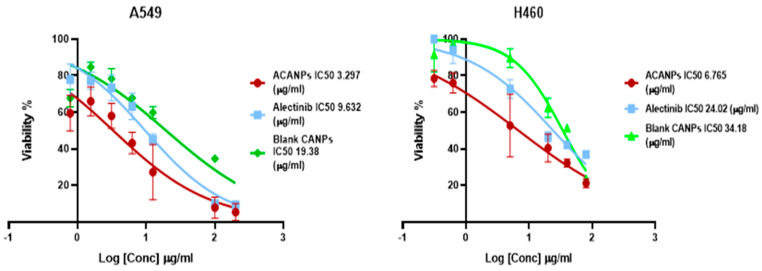
The cytotoxicity of ACANPs in A549 and H460 cell lines.

Alectinib-loaded nanoparticles consistently inhibited NSCLC cell growth more effectively than free Alectinib in both A549 and H460 lines (Figure 10). In A549 cells, ACANPs achieved an IC_50_ of 3.29 µg/mL vs. 9.63 µg/mL for the free drug. H460 cells similarly displayed a lower IC_50_ (6.77 µg/mL) with ACANPs compared to free Alectinib (24.02 µg/mL). Blank CANPs showed modest cytotoxic effects, suggesting that nanoparticle constituents alone can impact cell viability, likely through membrane interactions or oxidative stress. Overall, encapsulation improves intracellular drug delivery, enhancing Alectinib’s cytotoxic potency.

### 3.6. LC–MS/MS Method for Measurement of Alectinib in Rat Plasma

**Figure 11:**
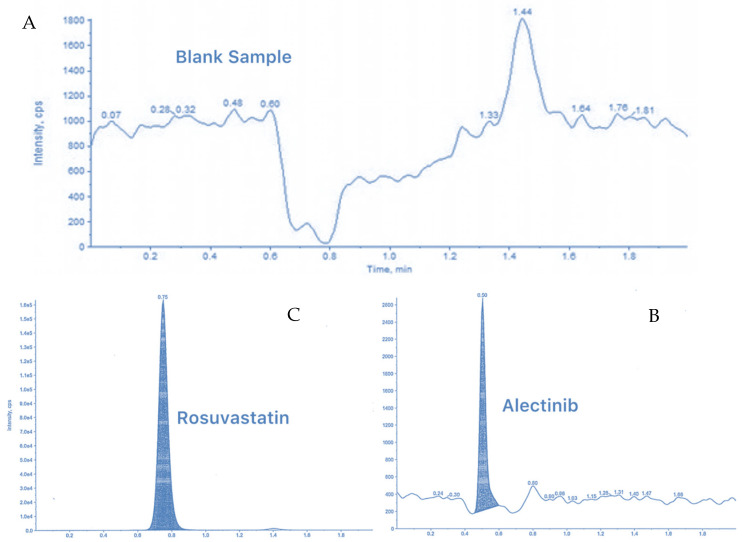
(**A**) LC-MS/MS chromatogram for the blank sample with no peak of Alectinib. (**B**) Alectinib at 483,300 Da, height: 2.48 × 10^3^ cps, RT: 0.504 min. (**C**) Rosuvastatin (internal standard) at 482,200 Da, height: 1.64 × 10^5^ cps, RT: 0.750 min.

The developed analytical method described in the methodology section was utilized to measure Alectinib concentrations in rat plasma samples. As shown in Figure 11, the chromatograms for blank plasma, Alectinib, and the internal standard Rosuvastatin collectively demonstrate the method’s efficiency. Alectinib exhibited a molecular weight of 483.3 Da and a retention time (RT) of 0.504 min. This method proved to be fast, economical, and time-efficient. Validation of the method was conducted in accordance with the FDA’s bioanalytical method validation guidelines, addressing key parameters including sensitivity, stability, linearity, and selectivity.

#### 3.6.1. Selectivity and Specificity (LC-MS/MS Method)

Selectivity and specificity tests confirmed the absence of interference from endogenous components, with clear and resolved peaks for Alectinib and the internal standard (Figure 11). The matrix peak area was less than 5% of the internal standard peak, meeting FDA guidelines.

#### 3.6.2. Linearity (LC-MS/MS Method)

**Figure 12:**
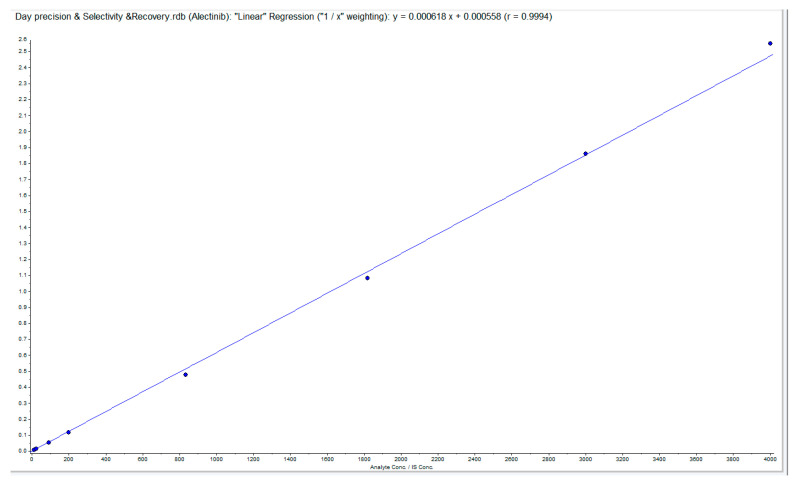
The LC-MS/MS linearity calibration curve with a correlation value (R^2^) of 0.9994.

The method showed linearity across a range of 10–4000 ng/mL of Alectinib in plasma, with a high correlation coefficient (R^2^ = 0.9994), indicating robust accuracy for pharmacokinetic studies (Figure 12).

#### 3.6.3. Precision and Accuracy (LC-MS/MS Method)

Precision and accuracy were assessed at four distinct concentration levels: LLOQ (10 ng/mL), low quality control (QC Low, 30 ng/mL), medium quality control (QC Medium, 1500 ng/mL), and high quality control (QC High, 3500 ng/mL).

The intra-day and inter-day precision results, indicated as the relative standard deviation (RSD%), remained within the acceptable threshold of 15% across all concentration levels, with RSD values of 6.15% for LLOQ, 4.50% for QC Low, 1.39% for QC Medium, and 2.83% for QC High (refer to Table S5). This validates the method’s dependability for repeated measurements.

The approach exhibited exceptional accuracy, with the measured concentrations being within the approved range of 85% to 115% of the nominal values. The accuracy values were 103.00% for LLOQ, 101.93% for QC Low, 106.72% for QC Medium, and 101.71% for QC High. The results demonstrate that the approach can deliver precise readings of Alectinib in plasma samples over an extensive concentration range.

The Lower Limit of Quantification (LLOQ) for the method was determined to be 10 ng/mL, exhibiting a relative standard deviation (RSD%) of 6.15%, thereby satisfying the FDA’s acceptance criterion of remaining within 20%. This illustrates the method’s sensitivity in identifying Alectinib at minimal concentrations, rendering it appropriate for pharmacokinetic investigations (Table S5).

#### 3.6.4. Recovery (LC-MS/MS Method)

The mean peak areas of the three extracted low, medium, and high quality control samples were compared to the mean peak areas of three pure reference solutions to assess Alectinib recovery in plasma (unextracted). The mean peak areas of extracted samples were compared to the mean peak areas of neat standard solutions (unextracted) of the same concentration to assess Rosuvastatin (IS) recovery. The recovery results were around 100% for Alectinib in human plasma, and all the replicates were acceptable, because the precision at each level was less than 15% (Table S6).

### 3.7. Pharmacokinetic Analysis

**Figure 13:**
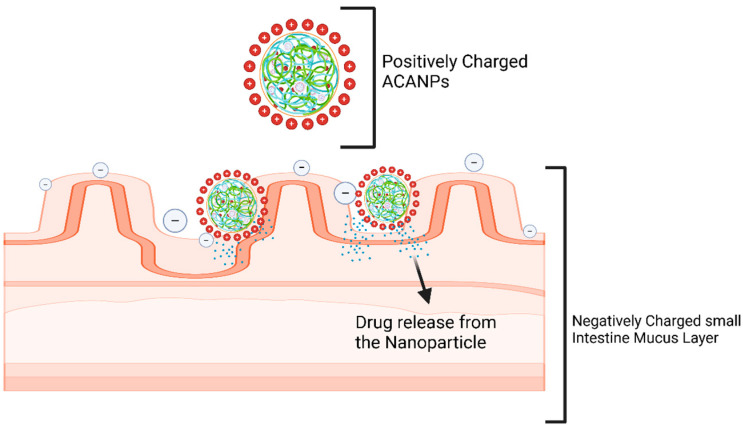
Interaction between positively charged ACANPs and the negatively charged mucus layer of the small intestine.

**Figure 14:**
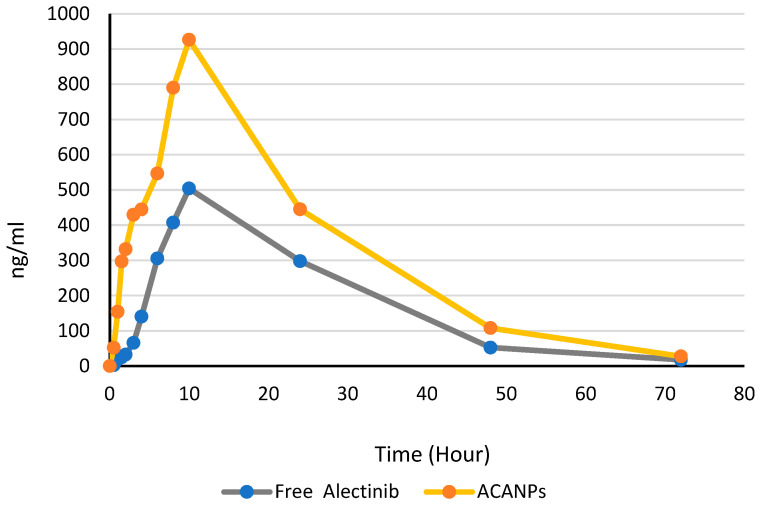
The plasma concentration–time profiles of Alectinib in the ACANP formulation and the free drug.

Alectinib-loaded chitosan–alginate nanoparticles leverage their distinct electrostatic properties to achieve a targeted and controlled drug delivery. The positively charged chitosan component electrostatically interacts with the negatively charged intestinal mucosa, improving adhesion and retention [33]. Chitosan and alginate also form a strong pH-sensitive degradation framework. This keeps nanoparticles stable in the stomach’s acidic environment, preventing the early release and degradation of Alectinib [34]. Once the nanoparticles reach the intestine’s neutral pH, alginate matrix erosion is regulated. This pathway facilitates the release of Alectinib, maintaining elevated systemic drug concentrations for therapeutic effectiveness. Figure 13 shows the proposed interaction between positively charged ACANPs and the negatively charged mucus layer of the membrane. Figure 14 shows the plasma profile of free Alectinib and Alectinib loaded on NPs.

The plasma concentration–time profiles offer important data regarding the pharmacokinetics of Alectinib across different dosage forms. Figure 14 illustrates the concentration profiles of Alectinib over time for both the nanoparticle-based formulation (ACANPs) and the free drug.

The pharmacokinetic parameters were assessed and compared between the two formulations to evaluate the influence of the nanoparticle formulation on drug release and bioavailability. Key parameters included maximum concentration (C_max_), time to reach maximum concentration (T_max_), area under the curve (AUC_0-72_ and AUC_0-inf_), elimination rate constant (Kel), half-life (t_1/2_), and percentage of extrapolated AUC, as presented in Table 6.

The findings demonstrate a notably elevated C_max_ for Alectinib-loaded nanoparticles (926.2 ± 166 ng/mL) in contrast to free Alectinib (504.1 ± 148 ng/mL), with both formulations reaching T_max_ at 10 h. The increase in C_max_ for the nanoparticle formulation indicates improved absorption or retention, likely due to the enhanced solubility and stability provided by the chitosan–alginate nanoparticle system. The same T_max_ indicated almost the same rate of absorption, which means that the release of Alectinib from the NPs was fast enough to mimic the free soluble Alectinib, while much more drug was absorbed.

The area under the curve (AUC_0−72_ and AUC_inf) values were significantly greater for the NP formulation, with the AUC_0−72_ measured at 23,049.13 ± 5420 ng·h/mL, in contrast to 12,907.65 ng·h/mL ± 3115 for the free drug, suggesting a higher systemic exposure for the nanoparticle formulation. The total AUC (AUC_0-inf_) increased to 23,528.78 ± 8269 ng·h/mL for the nanoparticles, compared to 13,216.80 ± 3917 ng·h/mL for the free drug, representing a 78% enhancement in bioavailability.

Both formulations demonstrated similar elimination rate constants (Kel), recorded at 0.05662 ± 0.00984 hr^−1^ for the free formulation and 0.05713 ± 0.00862 hr^−1^ for the nanoparticle formulation. The half-life (t_1/2_) was comparable, at roughly 12 h for both, suggesting that the encapsulation did not substantially affect the elimination process. The nanoparticle formulation exhibited a marginally lower percentage of extrapolated AUC (2.04%) in comparison to free Alectinib (2.34%), indicating a minimal contribution from extrapolated values and demonstrating the formulation’s stability throughout the dosing interval.

## 4. Conclusions

This study developed and characterized Alectinib-loaded chitosan–alginate nanoparticles (ACANPs) through a novel solid/oil/water (s/o/w) emulsification technique integrated with ionic gelation. The optimized formulation exhibited a high encapsulation efficiency, enhanced solubility, and improved release profiles, effectively addressing significant limitations related to the low bioavailability of Alectinib. Both in vitro and in vivo assessments demonstrated the effectiveness of the ACANPs, showing markedly improved anticancer activity and bioavailability relative to the free drug. The findings highlight the potential of chitosan–alginate nanoparticles as an effective platform for delivering poorly soluble anticancer agents such as Alectinib.

## Figures and Tables

**Table 1:** The encapsulation efficiency (EE %), ratios, solubilizing agents, and the method used of NP optimization.

Formulation Code	Amount of Alectinib (mg)	CS/ALG/TPP Ratio	Solubilizing Agent/Amount	Method	EE%
F1	5 mg	3:0:1	DMSO/1 mL	Ion Gelation	11 ± 2
F2	5 mg	4:0:1	DMSO/1 mL	Ion Gelation	4 ± 2.5
F3	5 mg	5:0:1	DMSO/1 mL	Ion Gelation	2 ± 1
F4	5 mg	5:0:1	SLS/10 mg	Ion Gelation	1.8 ± 1
F5	5 mg	5:0:1	PEG 400/1 drop	Ion Gelation	8 ± 4
F6	5 mg	5:0:1	Chremophore Rh 40/3 drops	Ion Gelation	20 ± 1
F7	5 mg	5:0:1	Chremophore Rh 40 4 drops	Ion Gelation	29 ± 5
F8	5 mg	5:0:1	Chremophore Rh 40 5 drops	Ion Gelation	31 ±2
F9	5 mg	5:1:1	Chremophore Rh 40 5 drops	Ion Gelation	35 ± 4
F10	5 mg	5:0:1	2 drops Tween 80	Ion Gelation	42 ± 5
F11	5 mg	5:0:1	3 drops Tween80	Ion Gelation	43 ± 3
F12	5 mg	5.0.1	2 drops Tween 80	S/O/W Emulsification with Ion Gelation	79 ± 4
F13	5 mg	5:1:1	2 drops of Tween 80	S/O/W Emulsification with Ion Gelation	97.1 ± 2

**Table 2:** Comparison of characteristic FTIR absorption bands of Alectinib, chitosan, alginate, and their physical mixture.

Functional Group	Alectinib (cm^−1^)	Chitosan (cm^−1^)	Alginate (cm^−1^)	Physical Mixture (cm^−1^)	Observation
N–H stretching	3454.51	~3400	~3400	~3410	Broad band retained, no major shift
C≡N stretching	2220	-	-	2118	Slight shift, reduced intensity
C–H aromatic stretching	3037.80	-	-	3035 (weak)	Retained
C=O (amide I, chitosan)	-	1651	-	1627	Minor shift, slight overlap
amide II (chitosan)	-	1560	-	1555 (broad)	Small shift
COO^−^ asymmetric (alginate)	-	-	1620	1597	Overlap with amide I
COO^−^ symmetric (alginate)	-	-	1417	1409	Minor shift
C–O–C/Glycosidic linkage	-	1150–1060	1028	1050–1030	Retained, small shift

**Table 3:** The particle size, PDI, and zeta potential of the prepared blank and loaded NPs.

Nanoparticle Composition	Particle Size ± SD (nm)	Zeta Potential ± SD (mV)	PDI ± SD
F13 (ACANPs)	161.1 ± 1.5	21.0 ± 0.32	0.233 ± 0.013
Blank CANPs	128.4 ± 3.2	10.0 ± 0.3	0.229 ± 0.011
Lyophilized ACANPs	161.9 ± 2.7	7.86 ± 0.4	0.296 ± 0.01

**Table 4:** Stability profile of ACANPs over 90 days (25 °C, dark glass container).

Time (Day)	Particle Size (nm)	Zeta Potential (mV)	PDI
0	0	0	0
1	161.1	21.0	0.233
7	160.5	20.9	0.219
14	165.2	21.1	0.248
30	163.2	21.6	0.246
60	166.6	21.2	0.244
90	156.2	22.9	0.227

**Table 5:** A model fitting of drug release.

Formula	Zero Order	First Order	Peppas	Higuchi	Hixon–Crowell
Free drug	0.972	0.890	0.797	0.990	0.972
ACANPs	0.849	0.979	0.923	0.970	0.895

**Table 6:** Pharmacokinetic parameters of free Alectinib and ACANP formulation.

Parameter	Free Alectinib	ACANPs
C_max_ (ng/mL)	504.1 ± 148	926.2 ± 166
T_max_ (hr) (median)	10	10
AUC_0–72_ (ng·h/mL)	12,907.65 ± 3115	23,049.13 ± 5420
AUC^0- inf^ (ng·h/mL)	13,216.80 ± 3917	23,528.78 ± 8269
Kel (h^−1^)	0.05662 ± 0.00984	0.057130 ± 0.00862
t_1/2_ (h)	12.24 ± 1.3	12.13 ± 1.5
% Extrapolated AUC	2.34%	2.04%

## Data Availability

All data are included in this manuscript and available according to the rules of the journal. Supplementary Materials are also available.

## Supplementary Materials

The following supporting information can be downloaded at: https://www.mdpi.com/article/10.3390/pharmaceutics17040492/s1, Figure S1: UV Absorbance Spectra of Blank Chitosan-Alginate Nanoparticles (CANPs) and Alectinib Hydrochloride (25 µg/mL) in Methanol. Figure S2: Particle Size Distribution, Polydispersity Index (PDI), and Zeta Potential Analysis of Alectinib-Loaded Chitosan-Alginate Nanoparticles (ACANPs; Formulation F13). Table S1: Main Liquid Chromatography and Mass Spectrometry Parameters for Alectinib Quantification. Table S2: Linearity Data of the Alectinib Calibration Curve Based on ICH Guidelines. Table S3: Intra-day Precision and Accuracy of Quality Control (QC) Samples Expressed as Relative Standard Deviation (RSD). Table S4: Recovery Results of Alectinib from Chitosan-Alginate Nanoparticle Matrix at Various QC Levels. Table S5: Accuracy and Precision Results of Alectinib Quantification in Rat Plasma at Different Concentration Levels. Table S6: Recovery Percentages of Alectinib in QC Samples Calculated at Low, Medium, and High Concentration Levels.
