# Supplementary material for: Alectinib-Loaded Chitosan–Alginate Nanoparticles: A Novel Synthesis Method with In Vitro and In Vivo Evaluations
Source: Pharmaceutics. 2025 Apr 8;17(4):492. doi: 10.3390/pharmaceutics17040492 (PMC12030190; doi:10.3390/pharmaceutics17040492)
Supplement: Supplementary file 1 [file pharmaceutics-17-00492-s001.zip › pharmaceutics-3549950-supplementary.pdf]

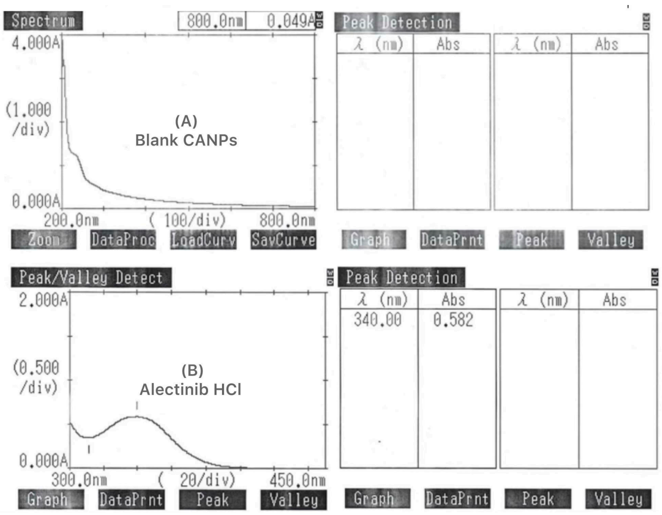

Figure S1 The UV scan of Blank CANPs & Alectinib HCl (25 µg/ml) in methanol.

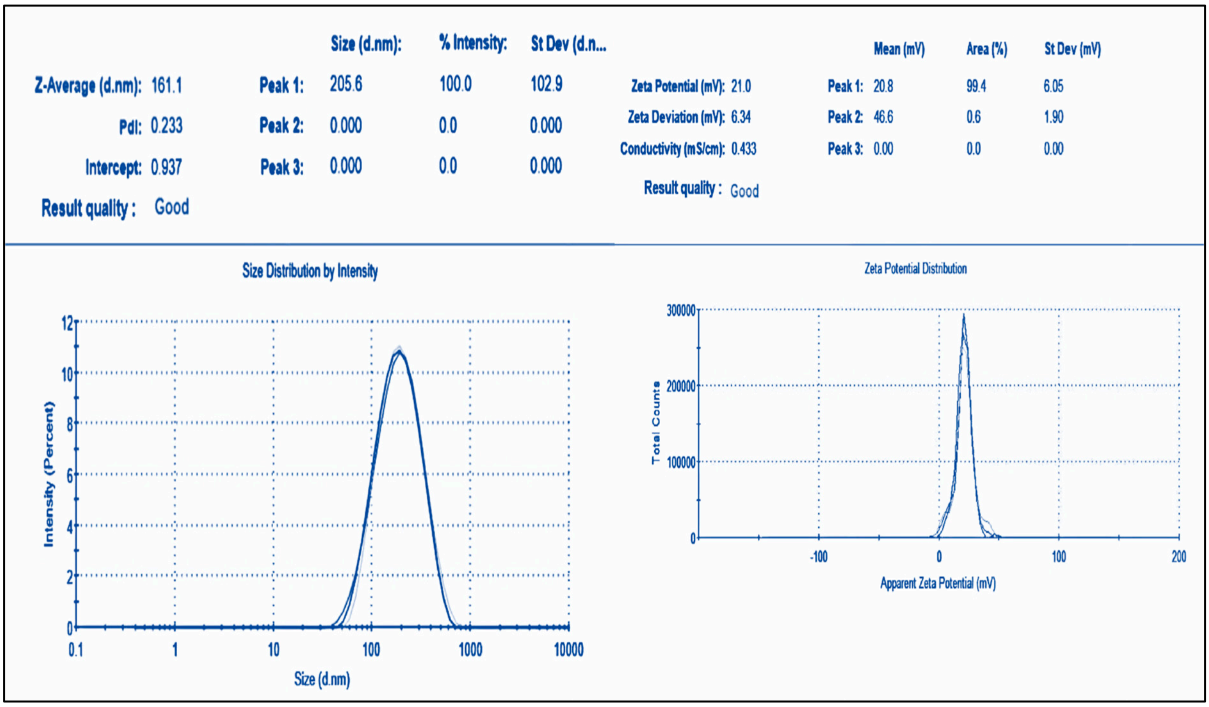

Figure S2 The particle size, PDI and Zeta Potential of ACANPs (F13).

Table S1 Main chromatographic and MS parameters.

Parameter	Condition/Setting
LC system	Agilent 1200 HPLC
Mass spectrometer	SCIEX 4000 triple quadrupole
Column	ACE C8 (5 µm, 4.6 × 50 mm)
Mobile phase	65% H_2O, 35% ACN, formic acid (1.0 mL/L)
Flow rate	1.0 mL/min
Column temperature	25°C
Autosampler temperature	5°C
Injection volume	10 µL
Runtime	2 min
Alectinib MRM (Da)	483.300 → 396.300
Rosuvastatin (IS) MRM (Da)	482.200 → 258.200

Table S2 The data of linearity of calibration curve of Alectinib based on ICH guideline.

Sample ID	Concentration µg/ml	Average Measured Area n = 3		
			STD	RSD
Cal 1	0.1953125	5958	33.620	0.564
Cal 2	0.390625	11444	83.193	0.727
Cal 3	0.78125	22280	88.097	0.395
Cal 4	1.5625	43297	73.921	0.171
Cal 5	3.125	87769	149.135	0.170
Cal 6	6.25	171568	316.633	0.185
Cal7	12.5	340546	305.491	0.090
Cal 8	25	681338	453.906	0.067
Cal 9	50	1352006	831.721	0.062
Cal 10	100	2696469	1059.077	0.039

Table S2 The precision and accuracy data QC samples and their RSD.

Sample ID	QC low (measured conc.)	QC low accuracy %	QC low RSD	QC mid (measured conc.)	QC mid accuracy %	QC mid RSD	QC high (measured conc.)	QC high accuracy %	QC high RSD
Replicate 1	5.24			52.182			92.738		
Replicate 2	5.161			52.074			92.348		
Replicate 3	5.144			52.209			92.581		
Replicate 4	5.133	103.202	0.782	52.066	104.186	0.229	93.464	102.949	0.481
Replicate 5	5.139			52.149			92.62		
Replicate 6	5.142			51.879			92.1761		
STDV	0.04			0.119			0.445		

Table S4 The recovery results of Alectinib from chitosan-alginate nanoparticles.

Concentration Level	Actual Concentration (µg/ml)	Recovered Concentration (µg/ml)	Average	% Recovery	RSD
QCLow	5.00	4.74<br>4.85<br>4.91	4.83	96.7	1.78
QCMid	50.00	50.81<br>50.23<br>50.76	50.60	101.2	0.64
QCHigh	90.00	88.32<br>89.27<br>89.41	89.00	98.9	0.67

Table S5 The results of precision and accuracy of Alectinib in rat plasma.

Sample	LLOQ (10 ng/mL)	QC Low (30 ng/mL)	QC Medium (1500 ng/mL)	QC High (3500 ng/mL)
Replicate 1	10.35	31.81	1616.24	3641.05
Replicate 2	9.72	31.93	1570.56	3720.46
Replicate 3	10.73	30.58	1628.85	3532.84
Replicate 4	9.53	29.32	1580.24	3455.65
Replicate 5	11.01	28.56	1610.56	3521.24
Replicate 6	10.98	31.25	1598.33	3487.51
Average	10.39	30.58	1600.80	3559.79
RSD%	6.15	4.50	1.39	2.83
Accuracy %	103.00	101.93	106.72	101.71

Table S6 The recovery percentage calculation for quality control (QC) samples at different concentration levels.

QC ID	QCs Area (Solution)	QCs Area (Extracted)	Average area (Solution)	Average area (Extracted)	Recovery (%)
QC LOW-1	42393	45347	42034	43701.83	103.9678
QC LOW-2	45623	46897			
QC LOW-3	40549	40869			
QC LOW-4	41251	42365			
QC LOW-5	41236	43159			
QC LOW-6	41152	43574			
QC MED-1	1936905	1943370	1857656	1837154	98.89638
QC MED-2	1867442	1758964			
QC MED-3	1793816	1824175			
QC MED-4	1857425	1764231			
QC MED-5	1936124	1933532			
QC MED-6	1754223	1798654			
QC High-1	3670235	3889907	3946175	3935438	99.72791
QC High-2	3673557	3863039			
QC High-3	4079356	3802904			
QC High-4	4062655	3905875			
QC High-5	4101469	4065149			
QC High-6	4089777	4085753			
